# Nrf2 Activation by 5-lipoxygenase Metabolites in Human Umbilical Vascular Endothelial Cells

**DOI:** 10.3390/nu9091001

**Published:** 2017-09-11

**Authors:** Nozomi Nagahora, Hidetoshi Yamada, Sayaka Kikuchi, Mayuka Hakozaki, Akira Yano

**Affiliations:** Iwate Biotechnology Research Center, 22-174-4 Narita, Kitakami, Iwate 024-0003, Japan; hyamada@ibrc.or.jp (H.Y.); s-fujisaki@ibrc.or.jp (S.K.); m-hakozaki@ibrc.or.jp (M.H.); akiray@ibrc.or.jp (A.Y.)

**Keywords:** Nrf2, reactive oxygen species, 5-lipoxygenase, human umbilical vein endothelial cells, 5-hydroxyeicosapentaenoic acid, 5-hydroxyeicosatetraenoic acid, 5-oxo-eicosatetraenoic acid

## Abstract

5-hydroxyeicosatetraenoic acid (5-HETE) and 5-hydroxyeicosapentaenoic acid (5-HEPE) are major metabolites produced by 5-lipoxygenase (5-LOX) from arachidonic acid (AA) and eicosapentaenoic acid (EPA). Effects of hydroxides on endothelial cells are unclear, although 5-LOX is known to increase at arteriosclerotic lesions. To investigate the effects of hydroxides on human umbilical vein endothelial cells (HUVECs), the cells were treated with 50 μM each of AA, EPA, 5-HETE, and 5-HEPE. Treatment of HUVECs with 5-HETE and 5-HEPE, rather than with AA and EPA, increased the nuclear translocation of NF-E2 related factor 2 (Nrf2) and upregulated the expression of heme oxygenase-1 and cystine/glutamate transporter regulated by Nrf2. Reactive oxygen species (ROS) generation was markedly elevated in HUVECs after treatment with 5-HETE and 5-HEPE, and the pretreatment with α-tocopherol abrogated ROS levels similar to those in the vehicle control. However, ROS generation was independent of Nrf2 activation induced by 5-HETE and 5-HEPE. 5-HETE was converted to 5-oxo-eicosatetraenoic acid (5-oxo-ETE) in HUVECs, and 5-oxo-ETE increased Nrf2 activation. These results suggest that 5-HETE works as an Nrf2 activator through the metabolite 5-oxo-ETE in HUVECs. Similarly, 5-HEPE works in the same way, because 5-HEPE is metabolized to 5-oxo-eicosapentaenoic acid through the same pathway as that for 5-HETE.

## 1. Introduction

Polyunsaturated fatty acids (PUFAs) and their metabolites have various functions in maintaining biological activities. Arachidonic acid (AA), eicosapentaenoic acid (EPA), and their metabolites, oxidized through enzymatic and non-enzymatic processes, are referred to as eicosanoids. Eicosanoids have been well studied and are known to work as mediators to control inflammation. Since Dyerberg et al. identified in 1978 that the abundance of AA and EPA in blood lipids was related to the incidence of atherosclerosis [1], several epidemiological studies have shown that intake of EPA could prevent cardiovascular diseases [2]. The mechanisms of action of EPA on the blood vessels have been elucidated using molecular biology approaches in vivo and in vitro [3]. Numerous studies have demonstrated that most of the eicosanoids produced from AA, such as leukotrienes, prostaglandins, and thromboxanes, accelerate blood coagulation and inflammatory reactions, while the eicosanoids produced from EPA suppress these processes due to the metabolites possessing lower activity than the metabolites of AA [4].

5-hydroxyeicosatetraenoic acid (5-HETE) and 5-hydroxyeicosapentaenoic acid (5-HEPE) are eicosanoids produced by 5-lipoxygenase (5-LOX), an enzyme mainly expressed in immune cells derived from the bone marrow [5]. As the first step, 5-LOX converts AA and EPA to 5-hydroperoxyeicosatetraenoic acid (5-HpETE) and 5-hydroperoxyeicosapentaenoic acid (5-HpEPE), respectively [6]. Subsequently, the hydroperoxides are metabolized to 4-series or 5-series leukotrienes by 5-LOX and the other enzymes. Simultaneously, cellular peroxidases, such as glutathione peroxidase, metabolize 5-HpETE to 5-HETE, and 5-HpEPE to 5-HEPE [7]. It has been reported that 5-LOX expression was upregulated at the site of atherosclerotic plaque and in patients with asthma [8,9]. 5-LOX is one of the therapeutic targets for treating inflammatory diseases, because 4-series leukotrienes produced from AA by 5-LOX aggravate inflammation [10]. Among the multiple studies on 5-LOX metabolites, the majority are on the effects of leukotrienes because of their potent bioactivity [6,9,11]. In contrast, the few studies on 5-HETE have shown that it promoted degranulation of platelet activating factor in human neutrophils [12], and it enhanced chemotaxis in polymorphonuclear leukocytes, while 5-HEPE did not have an effect [13]. The ligand activity of G-protein-coupled receptor 119 in pancreatic β cells and intestinal endocrine cells, as the positive activity of 5-HEPE, was reported [14]. Our group has also reported that peroxisome proliferator-activated receptors (PPARs) are activated by 5-HEPE in NIH-3T3 cells [15]. Although 5-HETE and 5-HEPE are produced in immune cells and are detected as the primary eicosanoids in the human blood [16,17], their bioactivities on endothelial cells remain unclear.

NF-E2 related factor 2 (Nrf2) is a key transcription factor regulating cellular detoxification and antioxidation. At the stationary state, Nrf2 is inactivated by binding to Kelch-like ECH-associated protein 1 (Keap1) in the cytoplasm, and the complex is readily degraded via the ubiquitin proteasome system [18]. Once the cell is exposed to stress, such as oxidative reactive oxigen species (ROS), ultraviolet, or electrophiles, Nrf2 dissociates from Keap1 and translocates to the nucleus. Nrf2 then binds to the antioxidant response elements (ARE), which induces the expression of genes encoding antioxidative proteins and enzymes, such as heme oxygenase-1. The activation of Nrf2 by proteasome inhibitors was reported to protect endothelial cells from oxidative stress [19]. In addition, it was shown that the expression of antioxidant enzymes in endothelial cells was enhanced through Nrf2 activation by 4-hydroxynonenal derived from AA and 4-hydroxyhexenal derived from docosahexaenoic acid [20,21]. These reports suggest that the activation of the Keap1-Nrf2 system in endothelial cells inhibits endothelial cell injury and prevents arteriosclerosis.

The clarification of the activities of 5-HETE and 5-HEPE in vascular endothelial cells is important because hydroxy fatty acids are produced as the major metabolites at the site of atherosclerotic plaque. The activities of most eicosanoids derived from AA are different from those derived from EPA; therefore, we assume that the activities of 5-HETE derived from AA and 5-HEPE derived from EPA may differ. The present study aimed to elucidate the effects of 5-HETE and 5-HEPE on endothelial cells from the viewpoint of Nrf2 and the differences in the effects of hydroxyl fatty acids. Thus, we investigated the effects of 5-HETE and 5-HEPE on Nrf2 activation in human umbilical vein endothelial cells (HUVECs) and compared these effects to those of AA and EPA.

## 2. Materials and Methods 

### 2.1. Reagents

AA and EPA were obtained from Nacalai Tesque (Kyoto, Japan). 5-HETE, 5-HEPE, and 5-oxo-ETE were purchased from Cayman Chemical (Ann Arbor, MI, USA). Both tertiary butylhydroquinone (tBHQ) and special grade methanol (MeOH) were purchased from Wako (Osaka, Japan). Alpha-tocopherol and tertiary butylhydroperoxide (TBHP) were obtained from Sigma-Aldrich (St. Louis, MO, USA) and Tokyo Chemical Industry Co., Ltd. (Tokyo, Japan), respectively.

### 2.2. Cell Culture

HUVECs and endothelial cell growth medium-2 (EGM-2), containing supplements, including vascular endothelial growth factor and antimicrobial reagents, were obtained from Lonza (Basel, Switzerland). Cells were cultured in EGM-2 at 37 °C in a humidified incubator supplied with 5% CO_2_. All experiments were performed using HUVECs at passages 4–7.

### 2.3. Immunocytochemical Analysis 

HUVECs were plated on poly-L-lysine coated cover glasses (13-mm diameter) in 24-well plates. After treatment with AA, EPA, 5-HETE, and 5-HEPE (50 μM each) at 37 °C for 3 h, cells were washed with phosphate-buffered saline (PBS) and fixed with 10% formalin solution (Wako) at room temperature for 10 min. Cells were permeabilized with 0.1% Triton-X 100 in PBS for 12 min, followed by blocking with 3% bovine serum albumin for 5 min. After incubation with mouse monoclonal anti-NRF2 antibody (clone 1F2; MBL, Nagoya, Japan) for 1 h, cells were incubated with donkey anti-mouse IgG H&L antibody (Alexa Fluor 488, Abcam, Cambridge, UK) for 30 min. Nuclear staining of cells was performed with 0.5 μg/mL propidium iodide (PI) solution (Dojido Laboratories, Kumamoto, Japan), and then the coverslips were mounted and observed using confocal laser microscopy with a FLUOVIEW FV1000 (OLYMPUS, Tokyo, Japan). The images were merged using FV10-ASW software. The experiments were performed three times and ten fields of view containing 25–30 cells per field were analyzed, and the green fluorescence intensity in the nucleus was quantified using image analysis software (Image J, 1.48 v, National Institute of Health, Bethesda, MD, USA).

### 2.4. Real-Time PCR

Type I collagen-coated 24-well plates were used, and the cells (1 × 10^5^ cells per well) were treated with 50 μM of AA, EPA, 5-HETE, and 5-HEPE, respectively, for 6 h. Total RNA was extracted from HUVECs using a NucleoSpin RNA kit (Macherey-Nagel, Düren, Germany), and the RNA was reverse-transcribed to cDNA with a PrimeScript RT reagent kit (Takara bio, Otsu, Japan). All procedures were conducted according to the supplier’s protocols. Real-time PCR analysis was performed with Fast SYBR Green master mix (Applied Biosystems, Foster, CA, USA) using the StepOne Plus real-time PCR system (Applied Biosystems, Foster, CA, USA). The PCR primers were designed as follows: hemeoxygenase-1 (HMOX1), forward primer: 5′-GGCCAGCAACAAAGTGCAAG-3′, reverse primer: 5′-TGGCATAAAGCCCTACAGCA-3′; cystine/glutamate transporter (SLC7A11), forward primer: 5′-GGCTGGTTTGAGCGAGTGTT-3′, reverse primer: 5′-TTGCAGAGAGTACATGGAGCC-3′; and 18S ribosomal RNA (18S, housekeeping gene), forward primer: 5′-TAAGTCCCTGCCCTTTGTACACA-3′, reverse primer: 5′-GATCCGAGGGCCTCACTAAAC-3′.

### 2.5. Detection of ROS

HUVECs were seeded on type I collagen-coated 24-well plates and were treated with EGM-2 containing fatty acid samples for 1 h after treatment with α-tocopherol for 1 h. CellROX Green (Thermo Fisher Scientific, Waltham, MA, USA) was added at a final concentration of 1 μM, followed by incubation at 37 °C for 1 h. After incubation, cells were washed twice with PBS and were then harvested using 0.25% trypsin-EDTA (Thermo Fisher Scientific, Waltham, MA, USA) and centrifuged at 1000× *g* for 3 min. The obtained pellets were washed with PBS twice and suspended in 1% foetal bovine serum in PBS. Then, the cells were stained with a PI solution (2 μg/mL final concentration) for 10 min to discriminate live cells from dead cells. Cell samples were analyzed using a flow cytometer (Cell Lab Quanta SC; Beckman Coulter, Brea, CA, USA).

### 2.6. Quantitative Analysis of 5-HETE, 5-HEPE, and 5-oxo-ETE in the Cells and Medium

HUVECs were seeded into 6-cm dishes (1 × 10^5^ cells per dish) and incubated with EGM-2 containing 50 μM 5-HETE, 50 μM 5-HEPE, or 5 μM 5-oxo-ETE for 6 h. After incubation, a portion of the medium was collected and mixed with three times the volume of acetonitrile containing 0.1% formic acid. The diluted media were centrifuged, and the supernatant was collected. Cells were washed twice with PBS and harvested using a scraper. The cell suspensions were centrifuged, and the pellets were sonicated with 0.1% formic acid in acetonitrile. 5-HETE, 5-HEPE, and 5-oxo-ETE were separated on an InertSustain ODS-3 column (2.0 mm diameter. × 250 mm; GL Science Inc., Tokyo, Japan) with gradient elution (10 mM ammonium acetate solution/acetonitrile, 55/45 to 5/95 in 25 min) at a flow rate of 0.2 mL/min. The compounds were identified and quantified by liquid chromatography-time of flight mass spectrometry (LC-TOFMS) (Agilent Technologies, Santa Clara, CA, USA) using Agilent Mass Hunter Workstation Software (version B.07.00 Service Pack 2, Agilent Technologies, Santa Clara, CA, USA). The velocity of drying gas was 10 L/min. The temperature of the gas was 325 °C. The voltages of the Vcap, fragmenter, and skimmer were 3500, 125, and 65 V, respectively. The pressure of the nebulizer was 30 psig.

### 2.7. Statistical Analysis

Data are expressed as mean ± standard deviation (SD). Statistical analysis was performed using the statistical software R [22]. One-way analysis of variance (ANOVA) with post-hoc Tukey’s test for multiple comparisons was used for statistical analysis. *p* values < 0.05 were considered statistically significant.

## 3. Results

### 3.1. Effects of 5-HETE and 5-HEPE on the Nuclear Translocation of Nrf2 and Gene Expression of Anti-Oxidative Enzymes in HUVECs

**Figure 1:**
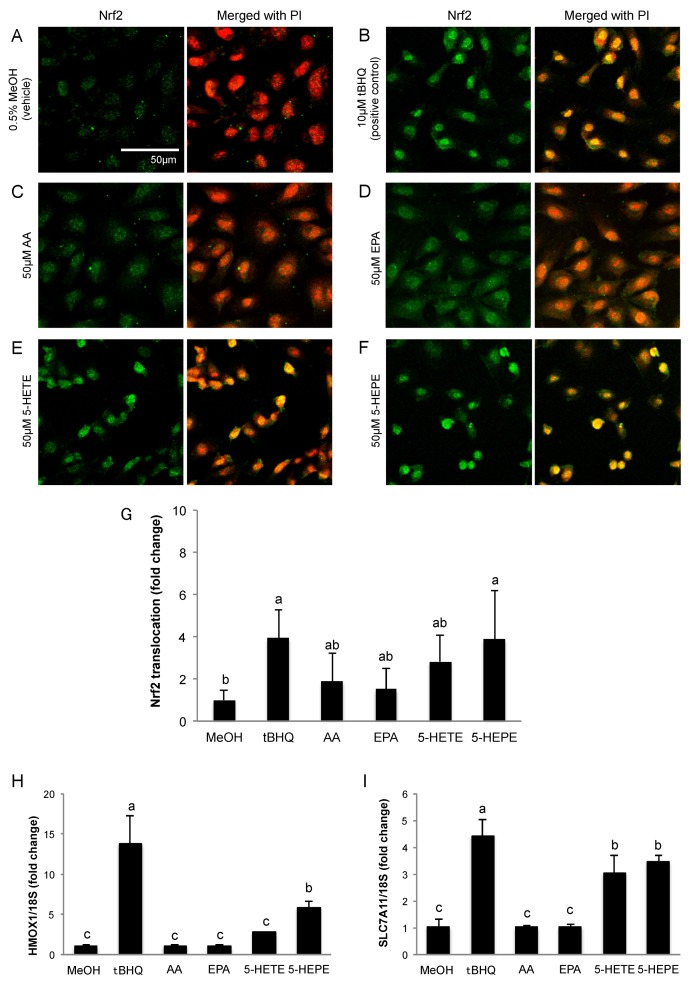
Effects of hydroxy fatty acids on Nrf2 translocation and expression of genes regulated by the Keap1-Nrf2 pathway in HUVECs. Nrf2 translocation was detected by immunostaining (green), and the nuclei were stained with PI (red). Cells were treated for 3 h with: 0.5% MeOH (vehicle) (**A**); 10 μM tBHQ (**B**); 50 μM AA (**C**); 50 μM EPA (**D**); 50 μM 5-HETE (**E**); and 50 μM 5-HEPE (**F**). The intensity of the green fluorescence signal in the nucleus was quantified using Image J (**G**). Data are expressed as the mean ± standard deviation (SD) (*n* = 10). Significant differences among the groups are indicated with different letters (one-way ANOVA followed by Tukey’s test, *p* < 0.05). Gene expression levels of: HMOX1 (**H**); and SLC7A11 (**I**) in HUVECs were analyzed and normalized to 18S gene expression levels (as an internal control) after incubation of each sample for 6 h. Data are expressed as mean ± SD (*n* = 4). Significant differences among the groups are indicated with different letters (one-way ANOVA followed by Tukey’s test, *p* < 0.05). Abbreviations: Nrf2, NF-E2 related factor 2; Keap1, Kelch-like ECH-associated protein 1; HUVEC, human umbilical vein endothelial cell; PI, propidium iodide; MeOH, methanol; tBHQ, tert-butylhydroquinone; AA, arachidonic acid; EPA, eicosapentaenoic acid; 5-HETE, 5-hydroxyeicosatetraenoic acid; 5-HEPE, 5-hydroxyeicosapentaenoic acid; ANOVA, analysis of variance; and HMOX1, heme oxygenase 1.

We determined the localization of Nrf2 in HUVECs by immunofluorescence staining. Nuclear translocation of Nrf2 in HUVECs was obviously facilitated by 10 μM tBHQ (Nrf2 activator) treatment in comparison to 0.5% MeOH treatment used as a vehicle control (Figure 1A,B,G). AA and EPA had no significant effects on Nrf2 translocation (Figure 1C,D,G). Treatment with 5-HETE slightly induced Nrf2 translocation (Figure 1E) but there was no significant difference when compared to 0.5% MeOH (Figure 1G). 5-HEPE treated cells showed significantly increased nuclear translocation of Nrf2 as compared to that of the vehicle control (Figure 1F,G). In addition, the expression levels of HMOX1 and SLC7A11, genes that are regulated by the Keap1-Nrf2 system, were examined (Figure 1H,I). After treatment with 10 μM tBHQ, HMOX1 and SLC7A11 expression levels were elevated in HUVECs. HMOX1 expression was significantly upregulated by 5-HEPE. Although there was no significant difference, 5-HETE elevated HMOX1 expression by 2.7-fold over treatment with 0.5% MeOH. SLC7A11 expression was upregulated by both 5-HETE and 5-HEPE. Neither EPA nor AA changed the expression of the HMOX1 and SLC7A11 genes.

### 3.2. ROS Generation Induced by EPA and Hydroxy Fatty Acids in HUVECs

**Figure 2:**
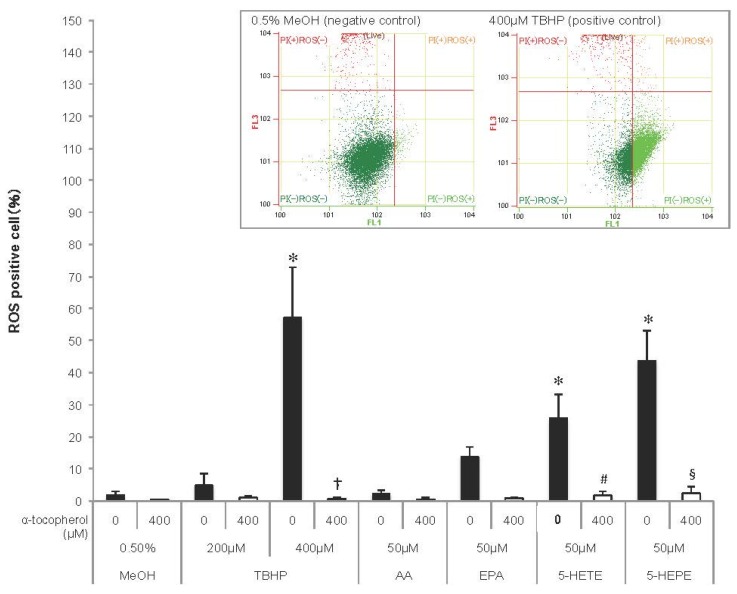
Reactive oxygen species (ROS) generation in HUVECs. Cells were incubated with 0.5% MeOH, tertiary butylhydroperoxide (TBHP), AA, EPA, 5-HETE, or 5-HEPE, for 1 h after treatment with or without 400 μM α-tocopherol for 1 h. ROS were detected using CellROX Green and measured with a flow cytometer, and the ratio of ROS positive cells to total live cell was represented as the percentage of ROS positive cells. Histograms of HUVECs treated with 0.5% MeOH (negative control) and 400μM TBHP (positive control) analyzed by flow cytometry are shown above the graph. Data are expressed as mean ± SD (*n* = 4). * *p* < 0.05 compared with 0.5% MeOH; † *p* < 0.05 compared with 400 μM TBHP; # *p* < 0.05 and § *p* < 0.05 compared with 50 μM 5-HETE and 50 μM 5-HEPE, respectively. Statistical analysis was performed with one-way ANOVA followed by Tukey’s test.

As ROS induce Nrf2 activation, ROS generation was detected in HUVECs using a fluorescent probe (Figure 2). TBHP, an ROS inducer, increased ROS generation in HUVECs in a concentration-dependent manner, and the increase was abolished by pretreatment with 400 μM α-tocopherol. ROS production tended to be enhanced in EPA-treated cells, but not in AA-treated cells. Both 5-HETE and 5-HEPE markedly increased ROS generation in HUVECs, and the ROS generation in 5-HEPE-treated cells was more than that in 5-HETE-treated cells. Pretreatment with 400 μM α-tocopherol suppressed the ratio of ROS positive cells treated with the hydroxy fatty acids to the same level as cells treated with 0.5% MeOH.

Effects of pretreatment with antioxidant on Nrf2 translocation and the expression of antioxidative enzymes in HUVECs treated with 5-HETE and 5-HEPE

**Figure 3:**
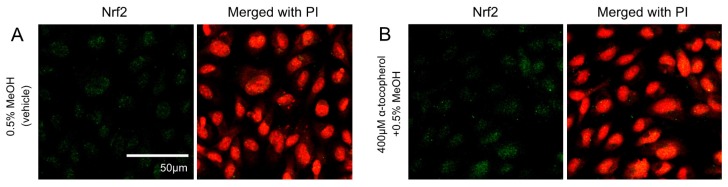

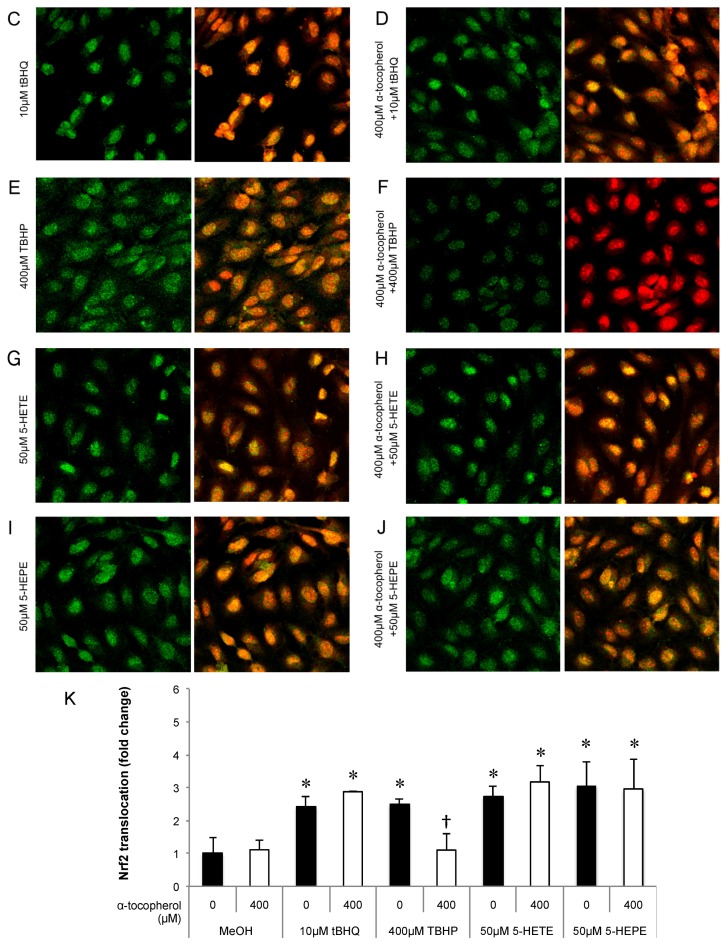
Effects of antioxidant treatment on nuclear translocation of Nrf2 in HUVECs. Nrf2 translocation was detected by immunostaining (green), and the nuclei were stained with PI (red). HUVECs were pretreated with or without 400μM α-tocopherol for 1 h and followed by incubated for 3 h with each samples: 0.5% MeOH (vehicle) (**A**); 0.5% MeOH with α-tocopherol (**B**);10 μM tBHQ (**C**); 10 μM tBHQ with α-tocopherol (**D**); 400 μM TBHP (**E**); 400 μM TBHP with α-tocopherol (**F**); 50 μM 5-HETE (**G**); 50 μM 5-HETE with α-tocopherol (**H**); 50 μM 5-HEPE (**I**); and 50 μM 5-HEPE with α-tocopherol (**J**). The intensity of the green fluorescence signal in the nucleus was quantified using Image J (**K**). Data are expressed as the mean ± SD (*n* = 8). The intensity of the green fluorescence signal in the nucleus was quantified using Image J (K). Data are expressed as the mean ± SD (*n* = 8). * *p* < 0.05 compared with 0.5% MeOH. Statistics analysis was performed with one-way ANOVA followed by Tukey’s test.

**Figure 4:**
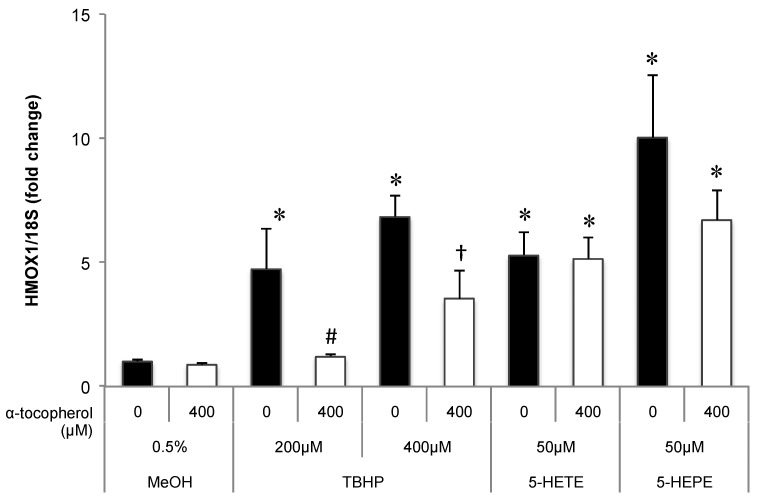
Effects of antioxidant treatment on HMOX1 upregulation by hydroxy fatty acids in HUVECs. Cells were pretreated with 400 μM α-tocopherol for 1 h; subsequently, 0.5% MeOH, 200 μM TBHP, 400 μM TBHP, 50 μM 5-HETE, or 50 μM 5-HEPE was added. After incubation with each sample for 6 h, gene expression of the cells was analyzed. Data are expressed as mean ± SD (*n* = 3). * *p* < 0.05 compared with 0.5% MeOH; # *p* < 0.05 compared with 200 μM TBHP; † *p* < 0.05 compared with 400 μM TBHP. Statistics analysis was performed with one-way ANOVA followed by Tukey’s test.

The contribution of ROS generation by 5-HETE and 5-HEPE on Nrf2 activation in HUVECs was investigated. Treatment of HUVECs with 0.5% MeOH did not influence the localization of Nrf2, and no change was noted when cells were pretreated with 400 μM α-tocopherol (Figure 3A,B). Nuclear translocation of Nrf2 was enhanced by 10 μM tBHQ, 400 μM TBHP, 5-HETE, and 5-HEPE; and pretreatment with α-tocopherol only suppressed the enhancement by 400 μM TBHP (Figure 3C–K). No inhibitory effect of α-tocopherol on HMOX1 expression was noted in HUVECs treated with 0.5% MeOH, while the upregulation of the gene induced by 200 μM and 400 μM TBHP was suppressed by antioxidant treatment (Figure 4). Pretreatment with α-tocopherol showed no effect on the increased HMOX1 expression mediated by 5-HETE and 5-HEPE (Figure 4).

### 3.3. Metabolism of 5-HETE and 5-HEPE in HUVECs

Metabolism of 5-HETE and 5-HEPE to oxo fatty acids has been reported in HUVECs [23]. To examine the metabolism and incorporation of 5-HETE and 5-HEPE in HUVECs, the concentrations of 5-HETE, 5-HEPE, and 5-oxo-ETE (the metabolite of 5-HETE) were determined in the medium and cells by LC-MS (Table 1). After 6 h of incubation of HUVECs with 50 μM 5-HETE, 54.691 ± 9.554 nmol/dish 5-HETE, 0.066 ± 0.039 nmol/dish 5-HEPE, and 3.072 ± 0.301 nmol/dish 5-oxo-ETE were detected in the medium. In the case of treatment with 50 μM 5-HEPE, 5-HEPE was detected in the medium at 38.886 ± 4.312 nmol/dish, but 5-HETE and 5-oxo-ETE were not detected. The medium contained 5-HETE (0.010 ± 0.009 nmol/dish) and 5-oxo-ETE (0.117 ± 0.010 nmol/dish) after incubating HUVECs with 5 μM 5-oxo-ETE. Uptake of 5-HETE into HUVECs incubated with 5-HETE resulted in a concentration of 0.377 ± 0.062 nmol/dish, and 5-HEPE (0.066 ± 0.039 nmol/dish) and 5-oxo-ETE (0.283 ± 0.053 nmol/dish) were detected in the cells. When HUVECs were treated with 5 μM 5-oxo-ETE, the cells incorporated 0.002 ± 0.000 nmol/dish 5-oxo-ETE and contained low levels of 5-HETE (0.005 ± 0.001 nmol/dish).

### 3.4. Effect of 5-oxo-ETE on Nrf2 Activation and ROS Generation in HUVECs

**Figure 5:**
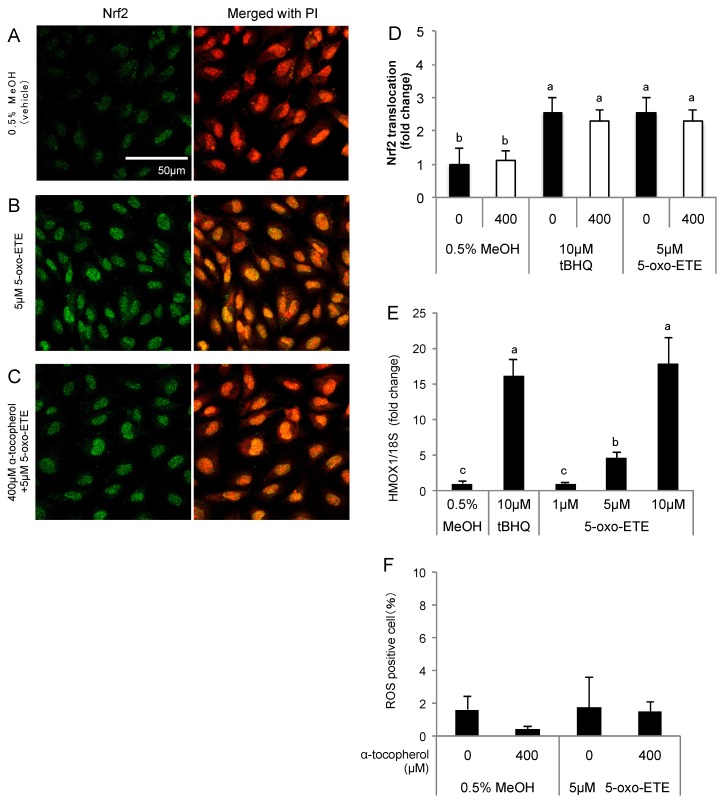
Effects of 5-oxo-ETE on Nrf2 activation in HUVECs. Nuclear translocation of Nrf2 was detected by immunostaining. The cells were treated for 3 h with: 0.5% MeOH (vehicle control) (**A**); and 5 μM 5-oxo-ETE (**B**); and the pretreatment with 400 μM α-tocopherol for 1 h was performed before treatment of 5-oxo-ETE (**C**). The intensity of the green fluorescence signal in the nucleus was quantified using Image J (**D**); Data are expressed as the mean ± SD (*n* = 8) Significant differences among the groups are indicated with different letters (one-way ANOVA followed by a post hoc Tukey’s test, *p* < 0.05). Gene expression of HMOX1 in the cells treated with 1, 5, and 10 μM 5-oxo-ETE for 6 h was analyzed (**E**). Data are expressed as mean ± SD (*n* = 3). Significant differences among the groups are indicated with different letters (one-way ANOVA followed by a post hoc Tukey’s test, *p* < 0.05). ROS generation in HUVECs was detected by CellROX Green (**F**). HUVECs were treated with 400 μM α-tocopherol for 1 h, and subsequently the cells were incubated for 1 h after the addition of 5 μM 5-oxo-ETE. Data are expressed as the mean ± SD (*n* = 4).

The effects of 5-oxo-ETE on HUVECs were examined as 5-oxo-ETE was detected in the media of HUVECs treated with 5-HETE. Nrf2 was translocated into the nucleus after treatment of HUVECs with 5 μM 5-oxo-ETE, and the translocation was not inhibited by α-tocopherol (Figure 5A–D). HMOX1 expression increased in a 5-oxo-ETE concentration-dependent manner (Figure 5E). 5-oxo-ETE, unlike 5-HETE and 5-HEPE, did not enhance ROS generation (Figure 5F).

## 4. Discussion

HUVECs were treated with samples at a concentration of 50 μM in the present study. Schuchardt et al. reported that serum concentrations of 5-HETE and 5-HEPE were 1.6 ± 0.13 nM and 0.33 ± 0.035 nM, respectively [17]. However, the concentration of 5-HETE and 5-HEPE at the inflammatory site, such as arteriosclerotic lesions that 5-LOX is induced [8] can be locally higher than that in the blood. We assumed the concentration of the hydroxyl fatty acids at the local site, and we performed the present experiments using 50 μM fatty acids that have no cell toxicity in vitro. 5-LOX is also expressed in HUVECs, but its expression is considerably low without stimulation such as cytomegalovirus infection [24]. It is thought that 5-HETE and 5-HEPE was hardly produced from AA and EPA by 5-LOX in the present experiments because HUVECs were treated with only AA or EPA.

In the present study, 50 μM 5-HETE and 5-HEPE activated the Keap1-Nrf2 pathway and upregulated the expression of not only HMOX1 and SLC7A11 but also thioredoxin reductase 1 (TXNRD1) and malic enzyme1 (ME1) (Figure S1), involved in maintaining the antioxidative capacity of the cells. The hydroxy fatty acids were also potent elicitors of ROS generation in HUVECs. Additionally, 5-HETE was metabolized to 5-oxo-ETE, which promoted nuclear translocation of Nrf2 without ROS generation. This is the first report revealing the effects of 5-HETE, 5-HEPE, and 5-oxo-ETE on Nrf2 activation in endothelial cells. It is known that AA-derived eicosanoids exert pro-inflammatory effects in contrast to EPA-derived eicosanoids [25]. Thus, the hypothesis of this study was that 5-HETE and 5-HEPE would have contrasting effects on HUVECs. However, our results revealed that there was no difference between the activities of the two hydroxy fatty acids.

A previous report showed that Nrf2 activation and HMOX1 expression increased after treatment of HUVECs with 25 μM EPA [26], while our present results indicated that HMOX1 expression was unchanged after treatment with 50 μM EPA (Figure 1H). It is known that EPA is easily oxidized because of multiple double bonds in the molecular structure. Gao et al. demonstrated that oxidized EPA activated the Keap1-Nrf2 pathway in HepG2 cells, and intact EPA at a concentration of 50 μM had no effect on that pathway [27]. According to the investigation by Majkova et al. [28], the binding of Nrf2 to the ARE of the NQO1 gene in porcine vascular endothelial cells increased after treatment with oxidized EPA. Ishikado et al. reported that treatment of HUVECs with 75 μM EPA slightly increased nuclear translocation of Nrf2 and HMOX1 expression [20]. Therefore, it is possible that the difference between our results and the previous report of Nrf2 activation by EPA might be caused by the oxidization state of EPA. In the present study, the purity of AA and EPA was checked by HPLC-photodiode array analysis before the experiments, and intact fatty acids were used for the cell treatments.

ROS are the one of the activating factors of Nrf2, and the antioxidative enzymes and proteins induced by Nrf2 activation scavenge excessive ROS in the cells. When HUVECs were treated with EPA, 5-HETE, and 5-HEPE, ROS levels were elevated (Figure 2). In previous reports, Zhang et al. [29] reported that EPA increase ROS in the HepG2 cell line, and Kang et al. [30] reported that DHA induce ROS in MCF-7 cells. Kang et al. proposed that ROS generation occurs through DHA peroxidation. However, the mechanism of ROS induction by n-3 PUFA is still unclear. Othman et al. demonstrated that the stimulation of retinal endothelial cells with 12-HETE increased subcellular ROS levels through the activation of NADPH oxidase [31]. 15-HETE induces ROS generation in pulmonary artery endothelial cells through upregulation of Nox4 expression, followed by the activation of p38 mitogen activated protein kinase (MAPK) [32]. Exogenous 20-HETE also generated ROS in pulmonary artery endothelial cells at a concentration of 1 μM via the activation of NADPH oxidase [33]. Similarly, it is possible that ROS generation by 5-HETE and 5-HEPE also might stimulate the intracellular ROS producing system, such as NADPH oxidase and the electron transport chain in mitochondria. Increases in ROS levels can stimulate the MAPK pathway [34], and the activation of the MAPK pathway can accelerate Nrf2 translocation through Nrf2 phosphorylation [35]. Pretreatment of α-tocopherol completely suppressed ROS generation induced by 5-HETE and 5-HEPE (Figure 2), but it did not abrogate Nrf2 activation (Figure 3 and Figure 4). These results suggest that Nrf2 activation by 5-HETE and 5-HEPE was independent of ROS generation by the hydroxy fatty acids. Further studies are necessary to elucidate the mechanisms underlying ROS induction and Nrf2 activation by 5-HETE and 5-HEPE.

5-HETE was metabolized to 5-oxo-ETE in HUVECs (Table 1), and this result is in agreement with the previous report by Erlemann et al. [23]. The conversion of 5-HETE to 5-oxo-ETE is catalyzed by 5-hydroxyeicosanoid dehydrogenase (5-HEDH) in immune and endothelial cells [23,36]. Similar to 5-HETE, the dehydrogenation of 5-HEPE to 5-oxo-eicosapentaenoic acid (5-oxo-EPE) is also catalysed by 5-HEDH [37]. Among eicosanoids, 5-oxo-ETE and 5-oxo-EPE are categorized as electrophilic ketone derivatives. Electrophilic reagents promote the nuclear translocation of Nrf2 by binding to cysteine residues of Keap1 [38]. Electrophilic fatty acids, such as 5-oxo-ETE and 5-oxo-EPE, also have been regarded as candidates for Nrf2 activators owing to their electrophilic properties [39,40], but the effects of 5-oxo-ETE and 5-oxo-EPE on the Keap1-Nrf2 pathway were uncertain until now. In this study, the exogenous supplementation of 5-oxo-ETE to HUVECs induced ROS-independent Nrf2 activation and 5 μM 5-oxo-ETE exerted the same effect as 50 μM 5-HETE (Figure 5A–C).

## 5. Conclusion

Our results suggest that 5-HETE and 5-HEPE have similar abilities to induce antioxidative enzymes in vascular endothelial cells by activating Nrf2 through their metabolites, such as 5-oxo-ETE and 5-oxo-EPE. However, ROS is generated by 5-HETE and 5-HEPE treatment at the concentrations at which Nrf2 activation occurred, which may negatively impact the cells. It was reported that 5-oxo-ETE had the ability to activate leukocytes, while 5-oxo-EPE had only 1/10th of the ability of 5-oxo-ETE [37]. Therefore, 5-oxo-EPE may be the more ideal Nrf2 activator candidate for maintaining blood vessel health in vivo because 5-oxo-ETE promotes the inflammatory reactions by activating leukocytes.

## Figures and Tables

**Table 1:** The concentrations of 5-HETE, 5-HEPE, and 5-oxo-ETE in the medium and HUVECs.

Cell (+)	Samples	5-HETE	5-HEPE	5-oxo-ETE
Medium (nmol/dish)	MeOH	N.D.	N.D.	N.D.
	5-HETE	54.691 ± 9.554	0.066 ± 0.039	3.072 ± 0.301
	5-HEPE	N.D.	38.886 ± 4.312	N.D.
	5-oxo-ETE	0.010 ± 0.009	N.D.	0.117 ± 0.010
Cell (nmol/dish)	MeOH	N.D.	N.D.	N.D.
	5-HETE	0.377 ± 0.062	0.002 ± 0.000	0.283 ± 0.053
	5-HEPE	N.D	0.418 ± 0.064	N.D
	5-oxo-ETE	0.005 ± 0.001	N.D.	0.002 ± 0.000

Cells were incubated with medium containing 0.5% MeOH, 50 μM 5-HETE, 50 μM 5-HEPE, or 5 μM 5-oxo-ETE for 6 h. Harvested cells and medium were deproteinized by acetonitrile containing 0.1% formic acid, and the supernatants were analyzed by LC-MS. Data are expressed as mean ± SD (*n* = 3). SD, standard deviation; 5-HETE, 5-hydroxyeicosatetraenoic acid; 5-HEPE, 5-hydroxyeicosapentaenoic acid; 5-oxo-ETE, 5-oxo-eicosatetraenoic acid; HUVECs, human umbilical vein endothelial cells; N.D., not detected.

## Supplementary Materials

The following are available online at www.mdpi.com/2072-6643/9/9/1001/s1, Figure S1: Gene expression of Malic enzyme 1 (ME1) and thioredoxin reductase 1 (TXNRD1) in HUVECs treated with eicosanoids for 6 h.
