# Supplementary material for: Nrf2 Activation by 5-lipoxygenase Metabolites in Human Umbilical Vascular Endothelial Cells
Source: Nutrients. 2017 Sep 11;9(9):1001. doi: 10.3390/nu9091001 (PMC5622761; doi:10.3390/nu9091001)
Supplement: Supplementary file 1 [file nutrients-09-01001-s001.pdf]

Supplementary Materials: Nrf2 Activation by 5-lipoxygenase Metabolites in Human Umbilical Vascular Endothelial Cells

Nozomi Nagahora, Hidetoshi Yamada, Sayaka Kikuchi, Mayuka Hakozaki and Akira Yano

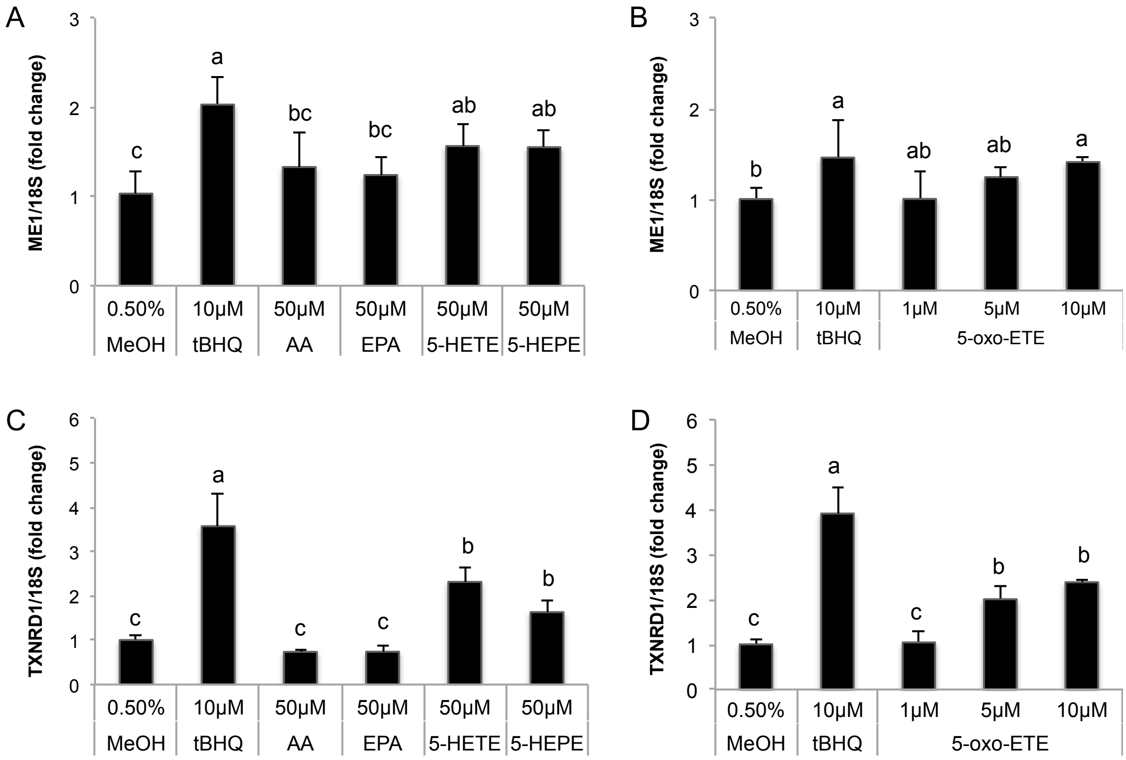

Figure S1. Gene expression of Malic enzyme 1(ME1) and thioredoxin reductase 1 (TXNRD1) in HUVECs treated with eicosanoids for 6 h. Effects of 0.5% MeOH (vehicle), 10 μM tBHQ (Nrf2 activator), AA, EPA, 5-HETE, or 5-HEPE (50μM) on ME1 expression (**A**), and effects of 1, 5, or 10 μM 5-oxo-ETE, on ME1 expression (**B**) was displayed. Effects of 0.5% MeOH, 10 μM tBHQ, AA, EPA, 5-HETE, or 5-HEPE (50μM) on TXNRD1 expression (**C**), and effects of 1, 5, or 10 μM 5-oxo-ETE, on TXNRD1 expression (**D**) was presented. Data are expressed as mean ± SD (n = 4). Significant differences among the groups are indicated with different letters (one-way ANOVA followed by a post hoc Tukey's test, $p < 0.05$).
